# Accumulation of MRI Markers of Cerebral Small Vessel Disease is Associated with Decreased Cognitive Function. A Study in First-Ever Lacunar Stroke and Hypertensive Patients

**DOI:** 10.3389/fnagi.2013.00072

**Published:** 2013-11-06

**Authors:** Marjolein Huijts, Annelien Duits, Robert J. van Oostenbrugge, Abraham A. Kroon, Peter W. de Leeuw, Julie Staals

**Affiliations:** ^1^Department of Neurology, Maastricht University, Maastricht, Netherlands; ^2^School for Mental Health and Neuroscience (MHeNS), Maastricht University, Maastricht, Netherlands; ^3^Cardiovascular Research Institute Maastricht (CARIM), Maastricht University, Maastricht, Netherlands; ^4^Department of Psychiatry and Psychology, Maastricht University, Maastricht, Netherlands; ^5^Department of Internal Medicine, Maastricht University Medical Centre, Maastricht University, Maastricht, Netherlands

**Keywords:** cerebral small vessel disease, cognition, white matter lesions, brain microbleeds, lacunar infarcts, enlarged perivascular spaces

## Abstract

**Background:** White matter lesions (WMLs), asymptomatic lacunar infarcts, brain microbleeds (BMBs), and enlarged perivascular spaces (EPVS) have been identified as silent lesions due to cerebral small vessel disease (cSVD). All these markers have been individually linked to cognitive functioning, but are also strongly correlated with each other. The combined effect of these markers on cognitive function has never been studied and would possibly provide more useful information on the effect on cognitive function.

**Methods:** Brain MRI and extensive neuropsychological assessment were performed in 189 patients at risk for cSVD (112 hypertensive patients and 77 first-ever lacunar stroke patients). We rated the presence of any asymptomatic lacunar infarct, extensive WMLs, any deep BMB, and moderate to extensive EPVS in the basal ganglia. The presence of each marker was summed to an ordinal score between 0 and 4. Associations with domains of cognitive function (memory, executive function, information processing speed, and overall cognition) were analyzed with correlation analyses.

**Results:** Correlation analyses revealed significant associations between accumulating cSVD burden and decreased performance on all cognitive domains (all *p* ≤ 0.001). Results remained significant for information processing speed (*r* = −0.181, *p* = 0.013) and overall cognition (*r* = −0.178, *p* = 0.017), after correction for age and sex. Testing of trend using linear regression analyses revealed the same results.

**Discussion:** We tested a new approach to capture total brain damage resulting from cSVD and found that accumulation of MRI burden of cSVD is associated with decreased performance on tests of information processing speed and overall cognition, implying that accumulating brain damage is accompanied by worse cognitive functioning.

## Introduction

Cerebral small vessel disease (cSVD) is a disorder affecting the small perforating vessels in the brain (Pantoni, 2010). cSVD is a strongly age-related disease and is one of the major causes of cognitive decline and functional loss in the elderly (Pantoni, 2010). It may result in clinically overt lacunar stroke syndromes, but also in clinically “silent” manifestations as white matter lesions (WMLs), asymptomatic lacunar infarcts, brain microbleeds (BMBs), and enlarged perivascular spaces (EPVS) (Wardlaw et al., 2006; Vermeer et al., 2007; Doubal et al., 2010; Pantoni, 2010).

Lesions due to cSVD are thought to disrupt frontal-subcortical circuits (Schmidtke and Hull, 2005) and consequently impair cognitive functioning. The abovementioned four markers of cSVD have all been individually linked to cognitive dysfunctioning (Werring et al., 2004; van den Heuvel et al., 2006; Carey et al., 2008; Zhu et al., 2010). However, these MRI markers of brain damage do not occur separately, and studies investigating the effect of the combined and accumulating presence of these individual markers on cognition are lacking. Recently, Wardlaw et al. (2013) suggested to search for methods to assess the total cSVD load on imaging in order to avoid over-reliance on one feature only.

Some studies focused on the combination of lacunar infarcts and WMLs (Baune et al., 2009; Jokinen et al., 2011). Baune et al. (2009) found a combined occurrence of lacunar infarcts and WMLs in 10% of the population and a significant difference between those patients with only one lesion type and patients affected by both on information processing speed and memory. The authors suggested that the combined occurrence was associated with stronger reductions in cognitive function than each of the two lesion types alone. Additionally, Jokinen et al. (2011) recently found that the relationship between progression of WMLs and new lacunes with cognitive decline was additive, but not synergistic.

No data are available on the clinical consequences of the total burden of “silent” cSVD on brain MRI, expressed by the co-occurrence of all four different markers of cSVD. The aim of this study therefore was to try a new method to capture the total brain damage caused by cSVD and investigate whether an accumulation of MRI markers of cSVD was associated with a decreased performance on cognitive function. We investigated this in patients at risk for silent cSVD, most probably due to hypertension-related occlusive arteriolosclerosis: patients with lacunar (small vessel) stroke and essential hypertensive patients.

## Materials and Methods

### Participants

Patients included in this study participated in two larger studies; the hypertensive patients in a longitudinal study on brain damage in hypertension (HYBRiD) (Henskens et al., 2009), whereas the lacunar stroke patients participated in a study on cognitive function after lacunar stroke.

Hypertensive patients were referred to our hypertension outpatient clinic of the Department of Internal Medicine of the Maastricht University Medical Center. Of 218 patients included, 112 patients completed a brain MRI and extensive neuropsychological assessment for this cross-sectional study. Exclusion criteria at baseline were a history of symptomatic cardio- or cerebro-vascular disease or contraindications for MRI.

Of 208 first-ever lacunar stroke patients presenting at the Neurology Department of the Maastricht University Medical Centre between February 2009 and July 2012, we consecutively included 77 patients. Lacunar stroke was defined as an acute stroke syndrome with a small ( <20 mm) ischemic lesion on acute brain MR in the brain stem, basal ganglia, or internal capsule, compatible with the occlusion of a single perforating small artery. If no such lesion was visible, we used established clinical criteria for lacunar stroke (de Jong et al., 2002). Patients with severe co-morbidity, either neurological or psychiatric, were excluded. Furthermore, patients without MRI or with possible other causes than cSVD (cardiac embolic source, cerebral large vessel disease, or carotid stenosis), were also excluded. Patients underwent neuropsychological assessment at 3 months after stroke to exclude acute phase effects.

The presence of vascular risk factors was recorded for all patients: hypertension, diabetes mellitus, hypercholesterolemia, and smoking. Data of vascular risk factors was missing for four hypertensive patients.

Both studies were approved by the Medical Ethics Committee of the Maastricht University Medical Centre and all participants gave written informed consent.

### Brain magnetic resonance imaging

MRI of the brain (1.5 or 3 T) was performed to obtain axial T2-weighted, fluid-attenuated inversion recovery (FLAIR) and T2*-weighted gradient echo images. Images were independently rated by two vascular neurologists (Julie Staals and Robert J. van Oostenbrugge) for the presence of asymptomatic lacunar infarcts, WMLs, BMBs, and EPVS for the first 137 patients. Kappas appeared to be satisfactory and the remaining images were rated by one vascular neurologist only. In case of disagreement or doubt, lesions were ascertained by consensus. Kappa’s for the presence of asymptomatic lacunar infarcts and deep BMBs were 0.62 and 0.63, respectively. Weighted kappa’s for dWMLs and EPVS in the basal ganglia were 0.77 and 0.69, respectively.

### Total burden of cSVD

We created an ordinal scale representing the total burden of cSVD, expressed by the co-occurrence of the different MRI markers. The presence of each of the four MRI markers for cSVD mentioned above (asymptomatic lacunar infarcts, WMLs, BMBs, and EPVS), was awarded with one point, resulting in a minimum of 0 and a maximum of 4.

#### Asymptomatic lacunar infarcts

We identified asymptomatic lacunar infarcts as sharply demarcated hyperintense lesions <20 mm on T2-weighted images with corresponding hypointense lesions with a hyperintense rim on FLAIR. In lacunar stroke patients, the lesion could not be compatible with the clinical stroke. One point was awarded when one or more asymptomatic lacunar infarcts were present.

#### White matter lesions

White matter lesions were graded according to Fazekas’ scale (Fazekas et al., 1987). One point was awarded in case of periventricular WMLs Fazekas score 3 (irregular hyperintensities extending into the deep white matter), and/or in case of deep WMLs Fazekas score 2 or 3 (confluent white matter hyperintensities). We used the Fazekas scores because they are histopathologically related to cSVD (Fazekas et al., 1993).

#### Brain microbleeds

Brain microbleeds were defined as punctate ( <10 mm) homogeneous foci of low signal intensity on T2*-weighted images. Symmetrical hypointensities in the globi pallidi were disregarded, since these most likely represent calcification. Since it was suggested that specifically deep BMBs (defined as basal ganglia, thalamus, and internal, external, and extreme capsule) are related to cSVD (Vernooij et al., 2008), one point was awarded only in case of the presence of one or more deep BMBs.

#### Enlarged perivascular spaces

Enlarged perivascular spaces were defined as round, oval, or linear shaped lesions with a smooth margin, absence of mass effect and with signal intensity equal to cerebrospinal fluid on T2-weighted images and (if visible) hypointense on FLAIR images without hyperintense rim to distinguish them from old lacunar infarcts (Bokura et al., 1998). Since EPVS at the level of the basal ganglia are specifically related to cSVD (Doubal et al., 2010), EPVS at this level were scored, on one slice and at one side in the most affected hemisphere only. The quantity of EPVS were rated as follows: category 1 = <10 EPVS, category 2 = 10–25 EPVS, and category 3 = >25 EPVS. One point was awarded in case of moderate (category 2) to extensive (category 3) EPVS.

### Assessment of cognitive function

Cognitive function was assessed by one trained neuropsychologist (MH) during a 2-h session. The test battery included the Rey Auditory Verbal Learning Test (RAVLT) (Brand and Jolles, 1985), Stroop Color Word Test (SCWT) (Golden, 1978), Trail making Test A and B (TMT) (Reitan, 1956), category and letter fluency (Luteyn, 1966; Lezak et al., 2004), and the following subtests of the Wechsler Adult Intelligence Scale III (WAIS III) (Wechsler, 2001), Symbol Substitution, Digit span, and Letter Number Sequencing. For the SCWT and TMT, interference scores were computed. Interference score SCWT = time on card 3 − Mean (card 1 + card 2). Interference score TMT = time card B − time card A.

We compared performances on different cognitive domains by using *Z* standard scores. A *Z*-score was calculated for each test score separately. For each domain we computed compound scores. Memory domain = (*Z*/RAVLT immediate recall + *Z*/RAVLT delayed recall + *Z*/RAVLT word recognition + *Z*/digit span forward)/4; Executive functions domain = (*Z*/SCWT interference + *Z*/TMT interference + *Z*/category fluency + *Z*/letter fluency + *Z*/digit span backward + *Z*/letter number sequencing)/6; Information processing speed = (*Z*/symbol substitution + *Z*/TMT A + *Z*/mean of Stroop card 1 and 2)/3. A compound score for overall cognitive function was calculated as the mean score of the three compound scores. *Z*-scores of tests with higher scores representing worse performance were inverted before computing the compound scores. Compound scores for memory, executive function, information processing speed, and overall cognition were missing for five, five, one, and nine patients, respectively. Therefore, analyses on these variables were performed with a different number of subjects.

### Statistical analysis

In the unadjusted analysis, we associated cognitive compound scores (memory, information processing speed, executive function, and overall cognition) with the levels of the total burden of cSVD, using bivariate correlation analyses. All analyses were done using the non-parametric spearman’s Rho due to the ordinal nature of the total cSVD burden quantification. In order to adjust for possible confounders, such as age and sex, we performed linear regression analyses with the confounding factors as independent and the cognitive domain as dependent variable. Residuals were saved and used in the non-parametric correlation analyses.

For testing of trend, the ordinal scale expressing the total burden of cSVD was considered as a continuous variable in a linear regression model with cognition as dependent variable, with adjustments for age and sex.

All analyses were performed using IBM SPSS statistics 18.0.

## Results

In total, 189 patients (112 hypertensive patients and 77 lacunar stroke patients) were included and 217 (86 hypertensive patients and 131 lacunar stroke patients) excluded. Excluded patients were older than those who were included (66.4 ± 15.0 vs. 59.9 ± 12.7 years, *p* < 0.001), but did not differ regarding sex (*p* = 0.095). Reasons for exclusion were: not interested (47.0%), atrial fibrillation (16.1%), cerebro-vascular accident or transient ischemic attack before inclusion (4.1%; for hypertensive patients only), pre-existent cognitive problems (3.2%), carotid stenosis (3.2%), contraindications for MRI (2.8%), death (1.8%), or other reasons (21.7%). Patient characteristics as well as the distribution of cSVD categories of the included lacunar stroke and hypertensive patients are shown in Table 1.

**Table 1:** **Patient characteristics and frequencies of cSVD extent**.

	All cSVD patients (*n* = 189)	Lacunar stroke patients (*n* = 77)	Hypertensive patients (*n* = 112)
Age, mean years (SD)	63.1 (14.3)	65.0 (12.0)	56.5 (12.1)
Male sex	108 (57.1)	43 (55.8)	65 (58.0)
Hypertension	159 (84.1)	47 (61.0)	112 (100.0)
Diabetes	9 (4.7)	7 (9.1)	2 (1.8)
Hypercholesterolemia	95 (51.6)	53 (69.7)	42 (38.9)
Current smoking	40 (21.3)	24 (31.2)	16 (14.4)
cSVD category
0	91 (48.1)	23 (29.9)	68 (60.7)
1	41 (21.7)	15 (19.5)	26 (23.2)
2	33 (17.5)	21 (27.3)	12 (10.7)
3	19 (10.1)	13 (16.9)	6 (5.4)
4	5 (2.6)	5 (6.5)	0 (0.0)

Data are shown in frequencies (%), except where reported otherwise.

### Total burden of cSVD

**Figure 1:**
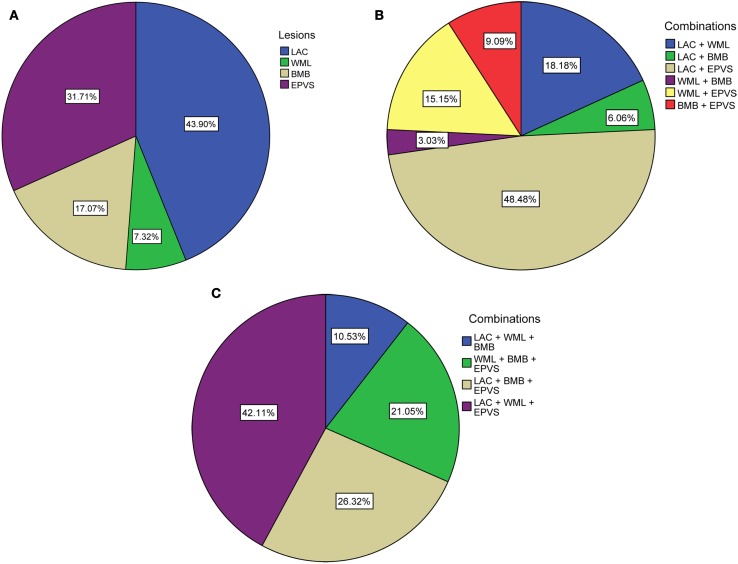
**Distribution of (combinations of) cSVD manifestations according to total burden of cSVD**. **(A)** Percentages of each lesion when one lesion is present. **(B)** Percentages of the combinations when two lesions are present. **(C)** Percentages of the combinations when three lesions are present. LAC, asymptomatic lacunar infarcts; WML, white matter lesions; BMB, brain microbleeds; EPVS, enlarged perivascular spaces.

Ninety-one patients (48.1%) had none of the silent MRI markers for cSVD, and five patients (2.6%) presented with all four markers. For patients with one, two, or three markers, the presence of the cSVD markers within each category is shown in Table 2 and the distribution of combinations is shown in Figure 1. Age increased significantly with increasing categories (0–4) of total burden of cSVD (54.4 ± 12.4; 60.6 ± 9.3; 65.5 ± 11.2; 70.6 ± 9.1; 77.8 ± 6.0 years, respectively, *p* < 0.001). Sex was not significantly different across the categories.

**Table 2:** **Distribution of cSVD manifestations according to total burden of cSVD**.

	Category 1 (*n* = 41)	Category 2 (*n* = 33)	Category 3 (*n* = 19)
Asymptomatic infarct	18 (43.9)	24 (72.7)	15 (78.9)
White matter lesions	3 (7.3)	12 (36.4)	14 (73.3)
Brain microbleeds	7 (17.1)	6 (18.2)	11 (57.9)
Enlarged perivascular spaces	13 (31.7)	24 (72.7)	17 (89.5)

Data are shown in frequencies (%).

### Association with cognitive function

**Figure 2:**
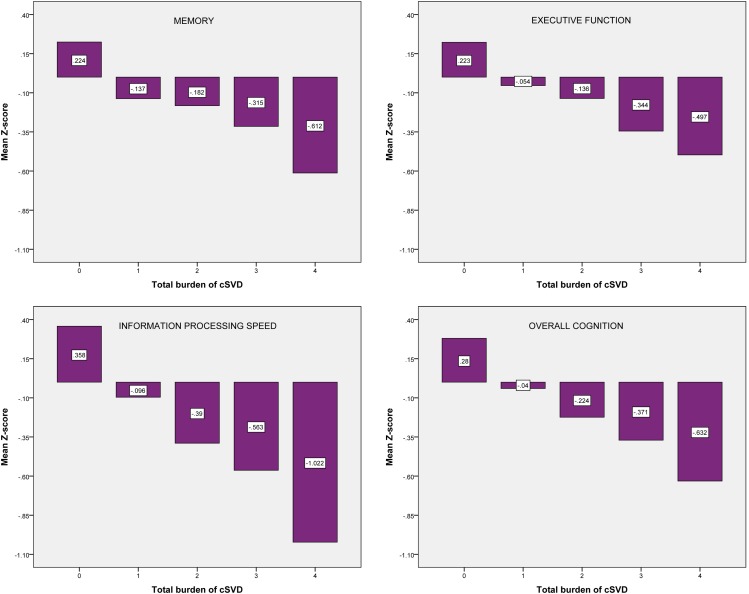
**Mean *Z*-scores on each of the cognitive domains, stratified by cSVD category**. cSVD, cerebral small vessel disease.

Figure 2 shows the mean *Z*-scores on each of the cognitive domains, stratified by cSVD category. Correlation analyses revealed significant negative correlations between cSVD category and all cognitive domains (all *p* < 0.001). When we adjusted for age and sex in the analyses, results remained significant for information processing speed and overall cognition (Table 3).

**Table 3:** **Correlation analyses between total burden of cSVD and cognitive function**.

	Model 1 correlation coefficient	Model 2 correlation coefficient
Memory	−0.333**	−0.108
Executive function	−0.312**	−0.129
Information processing speed	−0.438**	−0.181*
Overall cognition	−0.407**	−0.178*

Model 1: bivariate correlation analyses. Model 2: correlation analyses with adjustment for age and sex.

*p < 0.05.

**p < 0.001.

For the test of trend using linear regression analyses we found significant negative associations between cSVD category and all cognitive domains. When we adjusted for age and sex, results remained significant for information processing speed (β = −0.154; 95% CI = −0.254 to −0.054, *p* = 0.003) and overall cognition (β = −0.099; 95% CI = −0.183 to −0.016, *p* = 0.019). Results for executive function were borderline significant (β = −0.087; 95% CI = −0.181 to 0.007, *p* = 0.070).

Frequencies for the different combinations within each category were too small in order to compare cognitive function.

## Discussion

We demonstrated that accumulation of MRI markers of cSVD, expressed by the co-occurrence of these markers, is associated with decreased performance on all cognitive domains. After adjustments for age and sex, results remained significant for information processing speed and overall cognition.

The present literature mainly focused on the effect of single markers of cSVD on cognitive function, but no study has ever investigated the effect of the total burden of cSVD, expressed by all four MRI features that are now recognized as markers of cSVD, on cognitive function before. Our results extend the results of Baune et al. (2009) who found that the occurrence of two different MRI features (WMLs and lacunar infarcts) was associated with stronger reductions of cognitive function than the occurrence of one lesion only.

A recent study (Smallwood et al., 2012) proposed a pathology score for extent of cSVD and showed a relation between severity of cSVD pathology and cognitive impairment. From our results, it can be speculated that the co-occurrence of several different lesions represents more severe and extensive cSVD with more subcortical microstructural brain damage, resulting in cognitive dysfunction. However, the cross-sectional nature of our study prevents statements about causality. Longitudinal studies following increase of MRI markers and cognitive decline over time are needed in order to confirm a causal relationship between total burden of cSVD and cognitive function.

We do not know whether one silent MRI marker has more effect on cognition than the other marker. Regarding different combinations of cSVD markers, we were not able to compare cognitive function within each category of cSVD due to low frequencies. When higher numbers of patients can be included in future studies, it would be interesting to study whether one marker has a different effect weight than the other, or whether specific combinations of cSVD markers are associated with decreased cognitive performance more than others.

The major strength of this study is the extensive neuropsychological assessment which made it possible to evaluate cognitive function in several domains. The fact that the domain of information processing speed was most strongly and significantly related to cSVD, is in line with other studies that report information processing speed as one of the most affected cognitive domains in patients with cSVD (Prins et al., 2005).

Limitations of our study are mainly situated in the construction of the cSVD scale. We did not take into account all aspects, like the extent, location, or progression of each cSVD marker. Based on previous literature of cSVD (Vernooij et al., 2008; Doubal et al., 2010), we rated only deep BMBs and basal ganglia EPVS. However, we did not integrate for example the number of asymptomatic lacunar infarcts or number of BMBs, although it was recently found that a higher number of infarcts (Aggarwal et al., 2012) or BMBs (Poels et al., 2012) is associated with decreased cognitive performance. We dichotomized each marker which might lead to loss of power and information, and cut-off points may be arbitrary, although chosen with arguments. Nevertheless, according to a recent review by Wardlaw et al. (2013) on neuroimaging aspects of cSVD, approaches that try to capture the total cSVD burden on MRI are needed and we made a first attempt that is now open for further discussion, testing, and refinement. Another limitation is the variable MRI field strength that was used in our population. It was found that more BMBs can be detected at 3 T than at 1.5 T (Stehling et al., 2008) and this might have led to overrating in some of our patients. However, analyses with a correction for field strength did not change our results (results not shown). A final but important limitation is the mixed population of lacunar stroke and hypertensive patients, although both groups are at considerable risk of silent cSVD. The symptomatic stroke could have had a negative impact on cognitive function although we feel that the background silent cSVD is a more important determinant of cognitive function (Gottesman and Hillis, 2010).

In conclusion, we tested a new approach to capture total brain damage resulting from cSVD. We found that accumulation of MRI markers of cSVD is associated with decreased performance on tests of information processing speed and overall cognition, implying that accumulating brain damage is accompanied by worse cognitive functioning. We suggest further longitudinal studies in more homogeneous patient samples, and studies including more patients with higher prevalence of cSVD features in order to refine our cSVD scale and to be able to investigate whether specific combinations of cSVD markers are associated with decreased cognitive performance.

## Authors Contribution

Conception and design of the research: Marjolein Huijts, Annelien Duits, Robert J. van Oostenbrugge, Abraham A. Kroon, Peter W. de Leeuw, Julie Staals. Acquisition of data: Marjolein Huijts. Analysis and interpretation of the data: Marjolein Huijts, Annelien Duits, Robert J. van Oostenbrugge, Julie Staals. Statistical analysis: Marjolein Huijts. Drafting of the manuscript: Marjolein Huijts, Annelien Duits, Robert J. van Oostenbrugge, Julie Staals. Critical revision of the manuscript for important intellectual content: Marjolein Huijts, Annelien Duits, Robert J. van Oostenbrugge, Abraham A. Kroon, Peter W. de Leeuw, Julie Staals. Supervision: Annelien Duits, Robert J. van Oostenbrugge, Abraham A. Kroon, Peter W. de Leeuw, Julie Staals.

## Conflict of Interest Statement

The authors declare that the research was conducted in the absence of any commercial or financial relationships that could be construed as a potential conflict of interest.
